# Is there a difference in the expression levels of genes responsible for fluconazole resistance in *Candida albicans* isolated from people with different underlying diseases? A systematic review

**DOI:** 10.22034/cmm.2025.345313.1589

**Published:** 2025-03-03

**Authors:** Akbar Hoseinnejad, Mehrnoush Maheronnaghsh, Mojtaba Taghizadeh Armaki, Jalal Jafarzadeh, Mahnaz Fatahinia

**Affiliations:** 1 Student Research Committee, Ahvaz Jundishapur University of Medical Sciences, Ahvaz, Iran; 2 Department of Medical Mycology, School of Medicine, Ahvaz Jundishapur University of Medical Sciences, Ahvaz, Iran; 3 Department of Parasitology and Mycology, School of Medicine, Isfahan University of Medical Sciences, Isfahan, Iran; 4 Infectious Diseases and Tropical Medicine Research Center, Health Research Institute, Babol University of Medical Sciences, Babol, Iran; 5 Student Research Committee, Faculty of Medical Sciences, Tarbiat Modares University, Tehran, Iran; 6 Infectious and Tropical Diseases Research Center, Health Research Institute, Ahvaz Jundishapur University of Medical Sciences, Ahvaz, Iran

**Keywords:** *Candida albicans*, Fluconazole resistance, Gene expression, Systematic review, Underlying diseases

## Abstract

**Background and Purpose:**

Fluconazole is one of the primary antifungal agents in the treatment of candidiasis. However, long-term treatment and indiscriminate use of drugs from the azole family have created resistant isolates. *Candida albicans* cells can develop resistance to fluconazole through various mechanisms. The present study aimed to investigate the expression of genes involved in fluconazole resistance in *C. albicans* in people with different underlying diseases.

**Materials and Methods:**

Databases, such as PubMed, Scopus, and Web of Science were used to collect studies evaluating the expression levels of key *C. albicans* genes associated with fluconazole resistance from 1997 to 2024. Finally, 25 out of the 1,096 extracted studies were selected based on the inclusion and exclusion criteria.

**Results:**

This systematic review identified the genes encoding the ATP-binding cassette membrane pump (CDR1, CDR2) and the genes encoding the major facilitator superfamily pumps (MDR1), as well as the *ERG11* gene, are the most important effective genes in creating resistance of *C. albicans* to fluconazole. Based on the studies conducted since 1995, the CDR1 gene has the highest gene expression among the genes involved in resistance, followed by ERG11, MDR1, and CDR2, respectively.

**Conclusion:**

Comprehensive information about the activity of the genes and more studies on the genes involved in resistance, could provide valuable insights for further studies, prevent the occurrence of resistance to fluconazole and other azoles, and provide suitable treatments. The disease, as well as the dosage and duration of the antifungal therapy, may play an important role in determining the type of resistance mechanism of *C. albicans*. Therefore, further evaluation of the role of these genes in fluconazole-resistant species, along with their related gene products, is necessary.

## Introduction

Candidiasis is a primary or secondary fungal infection that can be caused by different species of *Candida*. It can present acutely, subacutely, or chronically on different human surfaces, such as skin, nails, mucous membranes, and internal organs. Mucosal candidiasis is one of the most common types of this infection, which mainly affects the mucous membrane of the mouth and esophagus; moreover, several species of *Candida*, especially *C. albicans*, can cause this disease [ 1 - 3 ]. For the treatment of this fungal infection, various antifungal drugs, such as azoles, especially fluconazole, are used. In recent years, numerous reports have highlighted the resistance of *C. albicans* to this antifungal in various patients, which ultimately caused the failure of the treatment in these cases [ 4 , 5 ].

**Figure 1:**
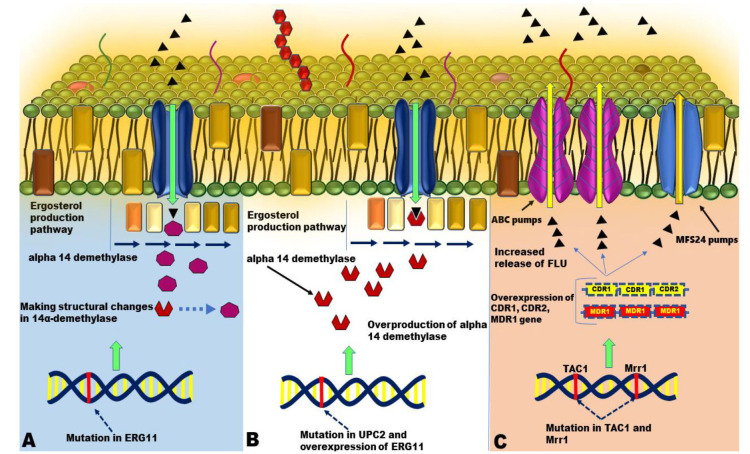
**A.** A mutation in the *ERG11* gene (encoding the 14-alpha-deamylase enzyme) results in an amino acid substitution in the 14-alpha-deamylase protein sequence. These changes lead to a reduction in the affinity of 14-alpha-deamylase for azoles or to structural changes that prevent fluconazole from reaching the active site. Ultimately, it causes a resistant phenotype. **B.** Mutation in *UPC2* and overexpression of *ERG11* followed by overproduction of alpha-14 demethylase and resistance to fluconazole. **C.** *TAC1* and *MRR1* genes control the expression of *CDR1*, *CDR2*, and *MDR1* genes. In addition, the mutation in each of them will increase the expression of *CDR1*, *CDR2*, and *MDR1*, and as a result, increase resistance against azole drugs.

Various mechanisms cause the resistance of *C. albicans* to fluconazole, which includes changes in 14α-demethylase, increased production of 14α-demethylase, exit phenomenon, and changes in the ergosterol synthesis pathway. One of the genes that play an important role in the resistance mechanisms against fluconazole is *ERG11*. This gene encodes the 14α-demethylase enzyme, and the mutation in this gene reduces the affinity of 14α-demethylase to fluconazole, and by making structural changes in 14α-demethylase, it does not allow azole drugs to stick to this enzyme, thereby leading to resistance in fungi (Figure 1-A)[ 6 - 8 ]. Mutations in the *UPC2* gene, which controls the expression level of the *ERG11* gene, can increase the expression of the *ERG11* gene and the number of its copies, eventually resulting in the overproduction of 14α-demethylase, which is considered another resistance mechanism to fluconazole in *C. albicans* (Figure 1-B) [ 9 - 10 ]. The *CDR1*, *CDR2*, and *MDR1*, which encode *Candida* membrane efflux pumps, are among the genes effective in resistance to azoles, especially fluconazole. The *CDR1* and *CDR2* encode the ATP-binding cassette (ABC) pumps, and *MDR1* encodes the major facilitator superfamily pumps, which ultimately cause the release of toxic molecules, such as azole drugs from *Candida* cells. *TAC1* and *MRR1* genes control the expression of *CDR1*, *CDR2*, and *MDR1* genes; in addition, the mutation in each of them will increase the expression of *CDR1*, *CDR2*, and *MDR1*, and as a result, increase resistance against azole drugs (Figure 1-C) [ 11 , 12 ].

**Figure 2:**
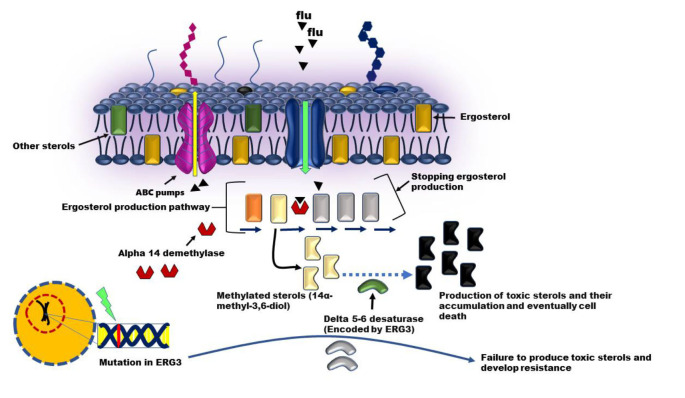
In the presence of fluconazole, inhibition of 14-alpha-deamylase leads to the accumulation of methylated sterols in the cell. These are then converted into toxic sterols by delta-5,6-desaturase (encoded by the ERG3 gene). If the ERG3 gene is mutated, these toxic products are no longer produced and the fungal cell survives and becomes resistant to fluconazole.

Another gene involved in the resistance of *Candida* to azoles is *ERG3*. It should be noted that azole drugs, including fluconazole, by inhibiting the 14α-demethylase enzyme, cause the accumulation of 14α-methyl-3,6-diol in the *C. albicans* membranes, and these methylated sterols are converted by the enzyme delta 5-6 desaturase (encoded by *ERG3*). It becomes toxic to the products, and the accumulation of these toxic substances causes the death of the fungus. When a mutation occurs in the *ERG3* gene, toxic products are not produced and *C. albicans* survive in the presence of fluconazole (Figure 2). This resistance mechanism has been shown experimentally by targeted deletion of the *ERG3* gene in laboratory strains [ 13 , 14 ].

Besides genetic mutations or expression changes, environmental factors, such as host immune status, biofilm formation, efflux pumps, and environmental stressors (oxidative stress and nutrient limitation) play significant roles in modulating fluconazole resistance in *Candida* species [ 15 ]. The present study aimed to investigate the expression of genes involved in *C. albicans* resistance in people with different underlying diseases. By obtaining comprehensive information about the activity of these genes, the emergence of resistance to fluconazole and other azoles can be prevented, and appropriate treatment methods can be identified.

## Materials and Methods

### Literature search

In this systematic review with a comprehensive search conducted by three authors, several databases, such as PubMed (Medline), Scopus, and Web of Science were used to collect all studies evaluating the expression level of the key involved in *C. albicans* resistance to fluconazole from 1997 to 2024. The search in the mentioned databases was performed using a specific set of medical subject headings ("Resistance mechanisms" OR resistant OR "molecular mechanisms" OR "Drug resistance" OR resistance OR "efflux pumps" OR "ABC transporter" OR "resistant phenotype" OR "Efflux Mechanisms" OR "ATP-binding cassette" OR "drug efflux" )  AND ( fluconazole* OR "antifungal agents" OR "antifungal drugs" OR azole OR "azole resistance" OR "Subinhibitory concentrations") AND ("*Candida albicans*" OR candidiasis OR "oropharyngeal candidiasis" OR "vulvovaginal candidiasis") AND (cdr* OR erg11* OR mdr1 OR "Gene expression" OR overexpression OR mrr1/2  OR erg-11) keywords from February 1 to 28, 2023. It should be noted that in addition to the articles selected for the study, the references of these studies and related articles were also searched. The present review only used articles that were published in English.

### Study selection criteria

The inclusion and exclusion criteria for the studies in this systematic review are as follows. All original English-language studies that were collected had three main criteria: 1) *C. albicans* isolates were collected from people with various underlying diseases associated with candidiasis, 2) the antifungal sensitivity of clinical *C. albicans* isolates to fluconazole was examined, and 3) the increased expression of fluconazole resistance genes in *C. albicans* isolates was investigated.

**Figure 3:**
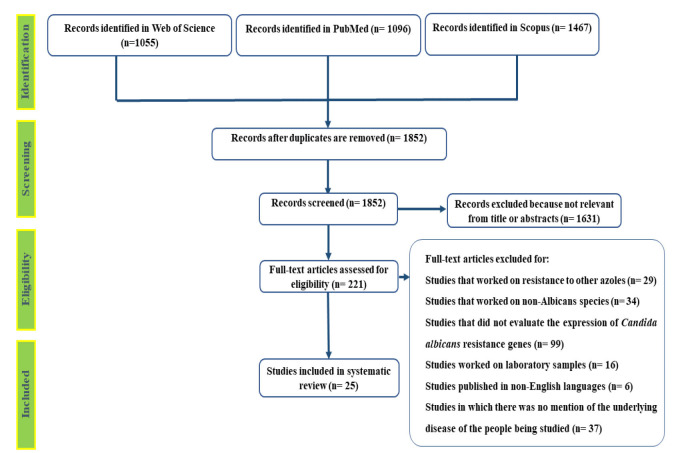
Flow diagram showing the study selection process.

Regarding the exclusion criteria, studies were excluded if they met any of the following conditions: 1) focused on laboratory and non-clinical *C. albicans* isolates, 2) were published in other languages, including Turkish, Persian, Chinese, and Spanish, and 3) examined only the mutations that occurred in fluconazole-resistant *C. albicans* isolates, 4) investigated the increased expression of genes responsible for resistance to other azoles, except fluconazole, 5) worked on animal samples, such as mice, and 6) were narrative reviews, systematic reviews, meta-analyses, best evidence reviews, short letters or reports, case studies, book reviews, and book sections. Finally, 25 studies were selected and reviewed completely (Figure 3).

This study was approved by the Ethics Committee of Ahvaz Jundishapur University of Medical Sciences Council (IR.AJUMS.REC.1402.197).

## Results

### Expression level of genes involved in C. albicans resistance to fluconazole in human immunodeficiency virus-infected patients

In recent years, the resistance of *C. albicans* to azole agents, particularly fluconazole, which is commonly used to treat oropharyngeal candidiasis in human immunodeficiency virus (HIV) patients, has resulted in treatment failure for these patients [ 41 ]. Based on the present research, 16 studies have been identified since 1995 that have examined the expression levels of genes responsible for fluconazole resistance in *C. albicans* isolated from HIV-infected patients.

Studies have revealed that most fluconazole-resistant *C. albicans* species are isolated from HIV-positive patients, many of whom have been on long-term fluconazole treatment during their therapy [ 42 , 43 ].

In HIV-infected patients, most fluconazole-resistant isolates exhibit lower levels of fluconazole intracellular accumulation than sensitive *C. albicans* strains. Among the studied genes, *CDR1/2* genes were found to be the most highly expressed in fluconazole-resistant *Candida* species isolated from HIV-positive patients, followed by *ERG11*, *MEDR1*, *BENr*, and *FLU1*. However, it is essential to mention that some of these genes did not have overexpression in several fluconazole-resistant isolates, and in some cases, their expression even decreased. This contradiction could be due to the existence of different mechanisms of fluconazole resistance in *C. albicans*, as well as differences in the *C. albicans* strains examined in different studies. In the reviewed studies, *CDR1* was found to be most significant in creating the resistant phenotype of *Candida* in HIV-positive patients (with an increase in expression from 2 to 17 times), followed by *CDR2*, *CDR3*, and *CDR4*, which were also investigated in some of these studies. However, unlike *CDR1/2* genes, they were not associated with *C. albicans* resistance to fluconazole [ 18 , 23 ]. Another gene found to be highly involved in causing resistance was *ERG11*. The *ERG11* gene was examined in 11 out of the 16 studies on *C. albicans* isolated from HIV-positive patients. In seven studies, there was an increase in the expression of this gene in resistant isolates [ 19 , 20 , 22 , 26 , 29 , 33 , 35 ], while in four studies the expression of *ERG11* was not increased in any of the resistant isolates [ 23 , 25 , 27 , 36 ]. The *MDR1* gene was not transcribed or barely transcribed in fluconazole-susceptible *C. albicans* strains. However, in most of the clinical isolates obtained from HIV-positive patients who were resistant to fluconazole, it was overexpressed, which eventually reduced the accumulation of fluconazole inside *Candida* cells and caused resistance to fluconazole. Some resistant strains of *C. albicans* in HIV-positive patients that did not overexpress *CDR1* extensively expressed another efflux pump gene, *BENr*, which plays an important role in resistance [ 16 ]. Another gene involved in the resistance isolates was the *FLU1* gene, which is a homologue of *MDR1*. Removal of this gene from several resistant *C. albicans* isolates increases their sensitivity to fluconazole. However, overexpression of this gene has not yet been suggested as a cause of fluconazole resistance in clinical isolates of *C. albicans* from HIV-infected patients [ 44 ].

The data from these 16 studies, conducted in different countries, such as the USA, Germany, and Japan, indicate that *CDR1* followed by *ERG11* genes play the most significant roles in creating the fluconazole-resistant phenotype. The genetic changes identified in this context suggest that the resistance of *C. albicans* to fluconazole develops in a stepwise manner and that one or more of these changes lead to the final resistant phenotype.

### Expression level of genes involved in C. albicans resistance to fluconazole in cancer patients

In total, 2 out of 25 collected studies investigated the expression level of genes responsible for fluconazole resistance in *C. albicans* isolated from cancer patients [ 39 , 40 ]. Based on these two studies, the increase in the expression level of *CDR1* and *CDR2* genes in *C. albicans* isolated from these patients can cause resistance of *C. albicans* to fluconazole. However, *CDR1* plays a greater role than *CDR2* in this resistance.

According to a study performed by Maheronnaghsh et al., *MDR1* is another gene found to be significantly over-expressed in fluconazole-resistant *C. albicans* isolated from cancer patients. Expression level of this gene is directly related to the level of resistance to fluconazole [ 39 ].

Maheronnaghsh et al. and Jahanshiri et al. noted that drugs used to treat certain cancers, such as cabazitaxel and idarubicin, which are also FDA-approved, have a negative effect on the antifungal activity of fluconazole. These drugs increase the expression of genes encoding drug-release proteins, such as *MDR1* or ABC (*CDR1* and *CDR2*), as well as the expression of the *ERG11* gene, and ultimately increase the resistance of *C. albicans* to fluconazole by 4 to 16 times. Usage of some chemical drugs in people suffering from various types of malignancies can cause the *C. albicans*-resistant phenotype in this category of patients by affecting the expression level of the above-mentioned genes.

### Expression level of genes involved in C. albicans resistance to fluconazole in organ transplant patients

According to Table 1, three studies have been conducted on *C. albicans* species isolated from transplant patients. In these individuals, as in HIV-infected and cancer patients, several genes play an essential role, and the combination of these genes leads to the emergence of *C. albicans* resistant phenotypes to fluconazole. In 2006, Xu et al. examined several resistant *C. albicans* species isolated from bone marrow transplant patients in terms of expression levels of resistance-causing genes. They found that *CDR* gene mRNA levels (including *CDR1* and *CDR2*), as well as some genes involved in drug resistance, such as *IFU5*, *RTA2*, and *IFD6*, had increased 2.5 to 7 times, compared to sensitive species.

**Table 1:** Increased expression in genes responsible for *Candida albicans* resistance to fluconazole in people with various underlying diseases

No.	Author, year, country, and reference	Underlying disease	Isolated source	Evaluation of the expression of *C. albicans* resistance genes	Overexpression in *C. albicans* resistance genes
1	Sanglard et al. (1995) Switzerland [ 16 ]	HIV-infected patients	Oral or esophageal samples	*CDR1*, *BENr*	*BENr* (FC: ND), *CDR1* (FC: ND)
2	White et al. (1997) USA [ 17 ]	HIV-infected patients	Oral samples	*CDR1*, *ELF1*, *ERG1*, *ERG7*, *ERG16*, *HST6*, *PRD1*, *YCF1*, *MDR1*	*ERG16* (FC: 4-5), CDR1 (FC: 5), *MDR1* (FC: ND)
3	Franz et al. (1998) Germany [ 18 ] HIV-infected patients	Oral samples	*CDR1*, *CDR2*, *CDR3*, *CDR4*	*CDR1* (FC: 4.6), *CDR2* (FC: 4 .6)
4	Lopez-Ribot et al. (1998) USA [ 19 ]	HIV-infected patients	Oral or esophageal samples	*ERG11*, *MDR1*, *CDR1*, *CDR2*	*ERG11* (FC: ND), *MDR1* (FC: ND), *CDR1* (FC: ND), *CDR2* (FC: ND)
5	Franz et al. (1998) Germany [ 20 ]	HIV-infected patients	Oral samples	*CDR1*, *CDR2*, *MDR1*, *ERG11*	*ERG11* (FC: ND), *MDR1* (FC: ND)
6	Maesaki et al. (1999) Japan [ 21 ]	HIV-infected patients	Oral or esophageal samples	*CDR1*, *MDR1*	*CDR1* (FC: ND)
7	Perea et al. (2001) USA [ 22 ]	HIV-infected patients	Oral samples	*CDR1*, *CDR2*, *MDR1*, *ERG11*	*CDR1* (FC: ND), *CDR2* (FC: ND), *MDR1*(FC: ND), *ERG11* (FC: ND)
8	Maebashi et al. (2001) Japan [ 23 ]	HIV-infected patients	Oral samples	*CDR1*, *CDR2*, *CDR3*, *CDR4*, *MDR2*, ERG-11	CDR1 (FC: ND), CDR2 (FC: ND)
9	Rogers et al. (2002) USA [ 24 ]	HIV-infected patients	Oral samples	*CDR1*, *CDR2*, *MDR1*, *ERG11*, *RTA3*, *FET34*, *FTR2*, *MIR1*, *ERG2*, *GPI1*, *CWH8*, *GPX1*, *IFD5*, *ALD5*, *MDH1*, *MET3*, *LYS21*, *CRD2*	*LYS21* (FC: 3.15), *MDH1* (FC: 3.52), *IFD5* (FC: 12.45), *CRD2* (FC: 4.6), *GPX1* (FC: 2.85), *CWH8* (FC: 3.6), *MIR1* (FC: 2.85), *CDR1*(FC: 3.67), *ERG2*(FC: 2.65)
10	Martinez et al. (2002) USA [ 25 ]	HIV-infected patients	Oral samples	*ERG11*, *MDR1*, *CDR1*, *CDR2*	*MDR1* (FC: ND), *CDR1* (FC: ND), *CDR2* (FC: ND)
11	Goldman et al. (2004) Brazil [ 26 ]	HIV-infected patients	Oral samples	*ERG11*, *MDR1*, *CDR1*, *CDR2*, *FLU1*	*ERG11* (FC: ND), *MDR1* (FC: ND), *CDR1* (FC: ND), *CDR2* (FC: ND), *FLU1* (FC: ND)
12	Ribeiro et al. (2005) Brazil [ 27 ]	HIV-infected patients	Vaginal samples	*ERG11*, *MDR1*, *CDR1*, *CDR2*	*CDR1* (FC: ND)
13	Xu et al. (2006) China [ 28 ]	Bone marrow transplantation patients	Blood culture samples	*IPF7530*, *YOR1*, *PXA1*, *ALD5*, *GRP1*, *SOD2*, *IPF10565*, *CRD1*, *CDR2*, *CTR1*, *CTR2*, *CCC2*, *FET3*, *PDR16*, *IFD6*,	*CDR1* (FC: ND), *CDR2* (FC: ND), *IFU5* (FC: ND), *RTA2* (FC: ND), *IFD6* (FC: ND)
14	Dunkel et al. (2008) Germany [ 29 ]	HIV-infected patients	Oral samples	*CDR1*, *CDR2*, *MDR1*, *UPC2*, *ERG11*	*CDR1* (FC: ND), *CDR2* (FC: ND), *MDR1* (FC: ND), *ERG11*(FC: ND)
15	Sikhala et al. (2010) Finland [ 30 ]	APECED patients	Oral samples	*CDR1*, *CDR2*, *TAC1*, *ERG1*, *MDR1*	*CDR1* (FC: 9.8), *CDR2* (FC: 20.4)
16	Mario et al. (2012) France [ 31 ]	HSCT recipient, heart transplant recipient	Respiratory tract, blood culture sample	*ERG11*, *MDR1*, *CDR1*, *ERG3*	*ERG11* (FC: 2.9 and 4.2) **
17	Sikala et al. (2013) Finland [ 32 ]	APECED patients	Oral samples	*CDR1*, *CDR2*,	*CDR1* (FC: ND), *CDR2* (FC: ND)
18	Morio et al. (2013) France [ 33 ]	HIV-infected patients	Oral, respiratory tract, intra-abdominal sample	*ERG11*, *MDR1*, *CDR1*,	*ERG11* (FC: ND), *CDR1* (FC: ND), *MDR1* (FC: 100)
19	Eddouzi et al. (2015) Tunisia [ 34 ]	Bone marrow transplantation patients	Oral swabs and blood culture samples	*ERG11*, *MDR1*	*ERG11* (FC: 2.7), *MDR1* (FC: ND)
20	Rosana et al. (2015) Indonesia [ 35 ]	HIV-infected patients	Oral samples	*ERG11*, *MDR1*, *CDR1*, *CDR2*	*CDR1* (FC: 1.03– 2.8), *CDR2* (FC: 1.4–121.1), *MDR1*(FC: 1.02–50.9), *ERG11* (FC: 1.7–133.4)
21	Salari et al. (2015) Iran [ 36 ]	HIV-infected patients	Oral samples	CDR1, CDR2, MDR1, ERG11	CDR1 (FC: ND), CDR2 (FC: ND), MDR1 (FC: ND)
22	Alizadeh et al. (2017) Iran [ 37 ]	Immunocompromised patients	Oral, vaginal, and cutaneous samples	*ERG11*	*
23	Hiyama et al. (2021) Japan [ 38 ]	Diabetics patients	Urine sample	*CDR1*, *CDR2*, *MDR1*, *ERG11*	*CDR1* (FC: 2.5), CDR2 (FC: 8.5), *MDR1*(FC: 22.5), *ERG11* (FC: 3.1)
24	Maheronnaghsh et al. (2022) Iran [ 39 ]	Cancer patients	Oral cavity and tongue samples	*ERG11*, *MDR1*, *CDR1*, *CDR2*	*CDR1* (FC: 1.79), *MDR1* (FC: 9.64)
25	Jahanshiri et al. (2022) Iran [ 40 ]	Head and neck cancer patients	Oral samples	*ERG11*, *MDR1*, *CDR1*, *CDR2*	*MDR1* (FC:18.34-24.06), *CDR1* (FC: 2), *CDR2* (FC: 2), *ERG11* (FC: 4.5-8)

FC: Fold Change, HIV: human immunodeficiency virus, APECED: Autoimmune polyendocrinopathy-candidiasis-ectodermal dystrophy, HSCT: Hematopoietic stem cell transplant

* : Genes without overexpression

** : Increased expression of resistance genes was measured in only two isolates. One showed a 2.9-fold increase, the other a 4.2-fold increase

It is important to point out that the IFU5 gene can increase the pathogenicity of *C. albicans* by interacting with the Efg1 gene. Moreover, *RTA2* genes increase the resistance of *C. albicans* to fluconazole, and the *IFD6* gene increases the ability of *C. albicans* to form biofilms [ 45 - 47 ]. The increase in *CDR* gene mRNA expression is mostly related to *CDR1*, and *CDR2* expression level did not show much difference between resistant and sensitive isolates. [ 28 ]. A study was conducted by Morio et al. in France on fluconazole-resistant *C. albicans* isolated from hematopoietic stem cell transplant recipients and heart transplant recipients. They found that among the *PMA1*, *ERG11*, *MDR1*, *CDR1*, and *ERG3* genes that were examined, only the *ERG11* gene showed increased expression in resistant isolates, compared to sensitive isolates [ 31 ].

These observations are consistent with the findings of a study performed by Eddouzi et al. (2015). In their study, Eddouzi, like Morio, found that the *ERG11* level in resistant isolates showed a significant increase. In the aforementioned study, in addition to the *ERG11* gene, the expression of the *MDR1* gene also increased in resistant isolates [ 34 ].

### Expression level of genes involved in C. albicans resistance to azole in diabetic patients

According to a study conducted by Hiyama et al., *C. albicans* resistance to fluconazole is significantly the result of increased expression of membrane current transporter genes. In contrast to *Candida* isolates from other underlying diseases, *MDR1* showed the highest increase in expression in *C. albicans*, compared to sensitive species, followed by *CDR2*, *CDR1*, and *ERG11* [ 38 ].

### Expression level of genes involved in C. albicans resistance to azole in autoimmune polyendocrinopathy-candidiasis-ectodermal dystrophy patients

In two studies carried out by Siikala et al. in 2013 and 2010 in Finland on resistant *C. albicans* isolates obtained from autoimmune polyendocrinopathy-candidiasis-ectodermal dystrophy (APECED) patients, *CDR1* and *CDR2* genes had higher expression levels. This increase in expression, compared to the wild-type species, is due to the presence of gain-of-function mutations in the regulatory factors of these three genes [ 30 , 32 ].

### Expression level of genes involved in C. albicans resistance to azole in immunocompromised patients

In another study conducted in Iran by Alizade et al. on immunocompromised patients, the level of *ERG11* gene expression was not significantly different between fluconazole-resistant and fluconazole-sensitive strains [ 37 ].

## Discussion

Fluconazole is considered one of the main antifungal drugs for the treatment of Candidiasis [ 37 ]. Despite this, long-term treatment and indiscriminate use of azole family drugs have created isolates resistant to these azoles. These resistant isolates are among the main problems in the treatment of Candidiasis [ 48 ]. Identification of the cause of resistance to azole drugs in clinical isolates of *C. albicans* can help to provide more appropriate treatment and prevent or control Candidiasis in the future [ 49 ]. *Candida albicans* cells can develop resistance to fluconazole at the molecular level through various mechanisms, which are explained in detail in the introduction section [ 22 , 50 , 51 ].

Based on the studies conducted since 1995, the *CDR1* gene has the highest expression level among the genes involved in resistance, followed by *ERG11*, *MDR1*, and *CDR2* genes, respectively. The *CDR1* gene has the highest expression in HIV-infected, cancer, and APECED patients, making *CDR1*-mediated resistance the most important resistance mechanism of *C. albicans* to fluconazole in these patients. However, in transplant patients, the *ERG11* gene has the highest expression, and the resistance mechanism in these patients is related to this gene. Moreover, the *MDR1* gene showed the highest increase in expression of the isolates obtained from diabetic patients. In fluconazole-resistant *C. albicans* isolated from HIV-positive patients, the development of resistance depends not only on continuous and intermittent administration of this drug but also on the cumulative dose of the received fluconazole (cumulative dose of fluconazole>10 g) [ 25 ].  Accumulation of this drug is prevented by *C. albicans* due to a significant increase in the expression level of *CDR1* and *BENr* genes. Both *CDR1* and *BENr* are multidrug transporter genes, each belonging to different classes of transporters [ 16 ]. 

In cancer patients, accumulation of chemotherapeutic drugs in *C. albicans* cells stimulates regulatory factors, increases *CDR1* and *MDR1* gene expression, and ultimately induces resistance. Since the increase in expression of these two genes in cancer and HIV-infected patients causes *C. albicans* resistance to fluconazole, the isolates in which the *MDR1* promoter is more active will grow under fluconazole or other selective pressure and survive longer than susceptible isolates. This provides numerous opportunities to create mutations that confer high levels of drug resistance [ 39 ]. A study of the combined effect of some FDA-approved oncology drugs with fluconazole found that a number of oncology drugs have a negative effect on antifungal activity.  Oncological drugs combined with azoles exacerbate antifungal resistance in *C. albicans* *in vitro*. These include tamoxifen, epirubicin, idarubicin, nilotinib, ceritinib, daunorubicin, cabazitaxel, and doxorubicin, which are prescribed for lymphoma, prostate cancer, bladder cancer and sarcoma [ 52 ].

Patients with type 2 diabetes are more likely to be affected by internal organ candidiasis [ 53 ]. An increase in the level of glucose, as well as an increase in the serum level of fructose, is one of the important causes of resistance of *C. albicansto* azole drugs in these patients [ 54 ]. Moreover, according to the study performed by Hiyama et al., an increase in urine glucose concentration promotes the growth of *C. albicans* and also significantly increases the expression of genes involved in *C. albicans* resistance to fluconazole, especially *CDR2* and *MDR1*. Increased expression of these two genes plays an important role in resistance to fluconazole in *Candida* species isolated from these patients [ 38 ].

In a study published in 2009, Uittamo et al. found that *C. albicans* isolated from APECED patients were able to produce high levels of carcinogenic acetaldehyde *in vitro*. In *C. albicans*, alcohol dehydrogenase, encoded by ADH1, catalyzes the oxidation of ethanol to acetaldehyde. Expression of *ADH1* is necessary for the production of ADH and acetaldehyde from ethanol [ 55 ]. According to the findings of a study performed by Sikala in 2013, the expression of the *ADH1* gene is strongly increased in isolates with low *CDR1* and *CDR2* expression levels. In other words, the expression levels of the *ADH1*, *CDR1*, and *CDR2* genes have an inverse relationship. Most fluconazole-resistant isolates of *C. albicans* have highly increased expression levels of *CDR1* and *CDR2*, while *ADH1* has the lowest expression level [ 32 ]. This important finding demonstrates the relationship between the acetaldehyde metabolism genes of *C. albicans* in these patients and azole resistance.

It is important to mention that in a number of studies, the overexpression of genes responsible for resistance has been observed only in some isolates and not in all isolates. Since the *ERG11* gene has been investigated in several studies, more aspects of this gene have been identified, compared to other major resistance-causing genes. It should be noted that fluconazole resistance in *C. albicans* is the result of different mechanisms controlled by different genes, with the *ERG11* gene being only one of them. One or more other genes may also be involved in creating the resistant phenotype.

Financial limitations and lack of advanced laboratory facilities in underdeveloped or less developed countries have led researchers to select fewer genes for study, and sometimes these selected genes do not show overexpression in both resistant and susceptible isolates. In this case, the resistance mechanism may be related to other genes that the researcher has not studied. For example, in a study conducted by Alizadeh et al. in Iran, only the *ERG11* gene was examined in resistant isolates of *C. albicans* isolated from immunocompromised patients. Finally, it was found that *ERG11* was not over-expressed in the resistant and sensitive isolates.

Extensive investigation of different genes involved in resistance, using molecular techniques, such as microarray and RNA-seq, can reveal new and broader aspects of their role. In isolates obtained from HIV-infected patients with candidiasis, the *CDR1* gene was the most highly expressed gene, while in transplant patients, the *ERG11* gene was the most highly expressed gene. This difference in resistance gene expression in isolates from patients with other underlying conditions, as mentioned in the results section, also exists and may have several potential causes.

## Conclusion

Genotypic differences between isolates from various parts of the world, the type of underlying disease, drug interactions between the antifungals and medications the individual was taking for the underlying condition, as well as the dosage and duration of the antifungals, may play an important role in determining the resistance mechanism of *C. albicans*. According to the conducted studies, it can be said that the expression level of fluconazole resistance genes varies in *C. albicans* species isolated from individuals with different underlying diseases. Nevertheless, to better clarify this issue, more studies need to be conducted with a larger number of fluconazole-resistant isolates and a larger number of genes.
